# Phylogeography of *Burkholderia pseudomallei* Isolates, Western Hemisphere

**DOI:** 10.3201/eid2307.161978

**Published:** 2017-07

**Authors:** Jay E. Gee, Christopher A. Gulvik, Mindy G. Elrod, Dhwani Batra, Lori A. Rowe, Mili Sheth, Alex R. Hoffmaster

**Affiliations:** Centers for Disease Control and Prevention, Atlanta, Georgia, USA

**Keywords:** phylogeography, Burkholderia pseudomallei, bacteria, single-nucleotide polymorphisms, SNPs, melioidosis, genome, Americas, Western Hemisphere, Puerto Rico, United States, Trinidad, Mexico

## Abstract

The bacterium *Burkholderia pseudomallei* causes melioidosis, which is mainly associated with tropical areas. We analyzed single-nucleotide polymorphisms (SNPs) among genome sequences from isolates of *B. pseudomallei* that originated in the Western Hemisphere by comparing them with genome sequences of isolates that originated in the Eastern Hemisphere. Analysis indicated that isolates from the Western Hemisphere form a distinct clade, which supports the hypothesis that these isolates were derived from a constricted seeding event from Africa. Subclades have been resolved that are associated with specific regions within the Western Hemisphere and suggest that isolates might be correlated geographically with cases of melioidosis. One isolate associated with a former World War II prisoner of war was believed to represent illness 62 years after exposure in Southeast Asia. However, analysis suggested the isolate originated in Central or South America.

Melioidosis is caused by the bacterium *Burkholderia pseudomallei* and is mainly associated with tropical areas. Although considered endemic to northern Australia and Southeast Asia, it has increasingly been recognized in other regions, such as Central America, South America, and the Caribbean (*1*–*3*).

A study by Pearson et al. based on multilocus sequence typing (MLST) data and analysis of single-nucleotide polymorphisms (SNPs) from whole genome sequences indicated that *B. pseudomallei* originated on the Australian continent because of the high level of genetic diversity seen in isolates from this region. They proposed that *B. pseudomallei* was transferred to Southeast Asia next, and from there was disseminated to other regions of the world (*4*).

Although MLST is the most common method to subtype isolates of *B. pseudomallei*, over time it has become recognized that it lacks the resolution to firmly link an isolate to a specific geographic origin (*4*–*7*). Because of homoplasy, the same sequence type (ST) can be found in isolates from different continents that are not truly closely related and instead have different genomic content (*7*).

To improve the ability to link genetic data with geographic provenance, we previously used a typing scheme for length polymorphisms in the 16S−23S internal transcribed spacer (ITS) of *Burkholderia* spp. to characterize isolates of *B. pseudomallei* with associations in the Western Hemisphere. We found that all isolates with a clear origin in the Western Hemisphere were ITS type G (*5*,*8*). We also established that the genomes contained the *Yersinia*-like fimbrial (YLF) gene, which is associated mainly with isolates not from Australia (*5*,*9*). Because of limited diversity detected by these methods, we hypothesized that a genetic bottleneck was associated with isolates from the Western Hemisphere (*5*).

Subsequent studies by Sarovich et al. (*10*) and Chewapreecha et al. (*11*) added support to this hypothesis by inferring relatedness from whole-genome SNPs. These studies indicate that Southeast Asia was the source of isolates from Africa, and Africa then became the source for *B. pseudomallei* in the Western Hemisphere, potentially associated with transfer to the Americas by the slave trade (*10*,*11*). Recent work has also shown the utility of SNP analysis in associating isolates of *B. pseudomallei* from patients with environmental isolates to establish epidemiologic links to better understand sources of infection (*12*–*14*).

In this study, we further characterized isolates associated with the Western Hemisphere by analysis of SNPs in whole-genome sequences to infer phylogenetic relatedness. We also sought to determine if this analysis could be used to associate isolates with geographic regions in the Western Hemisphere to improve epidemiologic associations, especially in cases where the source of infection was unclear.

## Materials and Methods

### Genomic DNA Extraction and Sequencing

DNA was extracted from isolates by using the Maxwell 16 Cell DNA Purification Kit with the Maxwell 16MDx automated nucleic acid purification system (Promega, Madison, WI, USA) per the manufacturer’s instructions. Final elution was performed by using PCR-grade water and RNase A. Samples were filtered by using a 0.1-μm filter. Sequencing was performed on an RSII apparatus (Pacific Biosciences, Menlo Park, CA, USA) for most isolates. DNA was sheared to 20 kb by passing it through a needle and used to generate 20-kb SMRTbell libraries by using a standard Template Preparation Kit (Pacific Biosciences). Libraries were further selected by size by using a BluePippin instrument (Sage Science, Beverly, MA, USA). The libraries were then sequenced by using the RSII apparatus (Pacific Biosciences), P6 polymerase, and C4 chemistry for 360-min movies. Library size ranged from 4 kb to 10 kb.

Sequence for MD2013 was determined from paired-end reads, which were generated by using an MiSeq apparatus (Illumina, San Diego, CA, USA). Genomic DNA was sheared to a mean size of 600 bp by using an LE220 focused ultrasonicator (Covaris Inc., Woburn, MA, USA). DNA fragments were cleaned by using Ampure (Beckman Coulter Inc., Indianapolis, IN, USA) and used to prepare single-indexed sequencing libraries by using the PrepX ILM DNA Library Preparation Kit (Wafergen Biosystems, Fremont, CA, USA), Truseq barcoding indices, and PCR primer cocktail (Illumina). Libraries were analyzed for size and concentration, pooled, and denatured for loading onto flow cells for cluster generation. Sequencing was performed by using Miseq 2 × 250-bp cycle paired-end sequencing kits (Illumina). After completion, sequence reads were filtered for read quality, base called, and demultiplexed by using Casava version 1.8.2 software (Illumina).

### Genome Assembly

De novo assembly was performed by using the Hierarchical Genome Assembly Process version 3 software (Pacific Biosciences) (*15*). Resulting consensus sequences were checked and edited for circularity either automatically by using Circlator (*16*) or manually by using Gepard (*17*). Illumina reads were cleaned by using BBDUK version 36.20 software (Joint Genome Institute, Walnut Creek, CA, USA) to remove PhiX (NC_001422.1). We used Trimmomatic version 0.36 software to clip off adapters and perform a 20-bp sliding window quality trim to a Phred 30 average (*18*). Cleaned reads were then assembled in SPAdes version 3.9.0 software by using the only-assembler option and retaining contigs >500 bp (*19*). Sequences were deposited into GenBank (accession numbers are in Table 1).

**Table 1:** Phylogeographic analysis of 26 isolates of *Burkholderia pseudomallei* from the Western Hemisphere by whole-genome sequencing*

Isolate	GenBank accession no.	Origin	Patient travel or residence	Year isolated	MLST type	ITS type
7894	CP018373,CP018374	Ecuador	Unknown	1960	11	G
724644	CP018377,CP018378,CP018379	Massachusetts, USA	Aruba	2012	698	G
CA2007	CP018418,CP018419	California, USA	Unknown	2007	518	G
CA2009	CP018380,CP018381	California, USA	Mexico	2009	95	G
CA2010	CP018382,CP018383	California, USA	Unknown	2010	550	C
CA2013a	CP018398,CP018399	California, USA	Unknown	2013	518	G
FL2012	CP018391,CP018392	Florida, USA	Trinidad	2012	297	G
GA2015	CP018416,CP018417	Georgia, USA	Panama and Peru	2015	436	G
IL2014	CP018414,CP018415	Illinois, USA	Mexico	2014	92	G
MD2013	MPSL01000000	Maryland, USA	Africa	2013	1053	C
MX2013	CP018395,CP018396,CP018397	California, USA	Mexico and Vietnam	2013	297	G
MX2014	CP018410,CP018411	California, USA	Mexico	2014	951	G
NY2010	CP018384,CP018385,CP018386	New York, USA	Aruba	2010	698	G
OH2013	CP018400,CP018401	Ohio, USA	None	2013	17	CE
PB 1007001	CP018387,CP018388	Arizona, USA	Costa Rica	2009	518	G
PR1982	CP018367,CP018368	Puerto Rico, USA	Unknown	1982	NA	G
PR1998	CP018369,CP018370	Puerto Rico, USA	Unknown	1998	92	G
PR2012	CP018393,CP018394	Puerto Rico, USA	Unknown	2012	297	G
PR2013a	CP018406,CP018407	Puerto Rico, USA, soil 1	None	2013	297	G
PR2013b	CP018408,CP018409	Puerto Rico, USA, soil 2	None	2013	297	G
RI2013a	CP018402,CP018403	Rhode Island, USA	Guatemala	2013	1038	G
RI2013b	CP018404,CP018405	Rhode Island, USA	Guatemala	2013	1038	G
Swiss2010	CP018389,CP018390	Switzerland	Martinique	2010	92	G
TX2004	CP018375,CP018376	Texas, USA	Southeast Asia	2004	297	G
TX2015	CP018412,CP018413	Texas, USA	Mexico	2015	92	G
VEN1976	CP018371,CP018372	Venezuela	Unknown	1976	12	G

*ITS, internal transcribed spacer; MLST, multilocus sequence typing; NA, not available (allele for *gltB* was not resolvable by conventional Sanger sequencing or whole-genome analysis).

### Strain Typing

We performed MLST as described by using traditional Sanger methods or genome assembly to query the *B. pseudomallei* MLST database (http://pubmlst.org/bpseudomallei/) (*5*,*6*,*20*–*23*). We used kSNP version 3.021 software to analyze SNPs for strains sequenced for this study (Table 1) and a reference panel of genomes (Table 2) (*24*). The maximum-parsimony tree from core SNPs was used to identify clades by using FigTree version 1.4.0 (http://tree.bio.ed.ac.uk/software/figtree/). Select isolates and clades were investigated for where SNPs occurred by manual interrogation of the kSNP version 3.021 variant call format output file and SnpEff version 4.3 (*25*).

**Table 2:** Reference genomes of *Burkholderia pseudomallei* strains from the Western Hemisphere used for phylogeographic analysis

Strain	Origin	GenBank accession no.
576	Thailand	ACCE01000000
350105	Hainan, China	CP012093, CP012094
1026b	Thailand	CP002833, CP002834
11–1617	Madagascar	SRR3145392
11–1696	Madagascar	SRR3145393
1258a	Thailand	AHJB01000000
1710b	Thailand	CP000124, CP000125
406e	Thailand	AAMM02000000
4900CF	Brazil	ARZE01000000
A79C	Papua New Guinea	JQHQ01000000
ABCPW1	Australia	JQIJ01000001
BCC215	Brazil	ABBR01000000
BEL2013	Madagascar	SRR3145396
BF103	Burkina Faso	SRR3145394
BF111	Burkina Faso	SRR3145395
Bp1651	California, USA, by way of Australia	CP012041, CP012042
Bp22	Singapore	AFBJ01000000
BPC006	China	NC_018527, NC_018529
K96243	Thailand	NC_006350, NC_006351
MSHR1655	Australia	AAHR02000000
MSHR305	Australia	CP006469, CP006470
MSHR3709	Australia	JRFK01000000
MSHR5107	Malaysia	JZXP01000001
MSHR5613	Australia	JQDK01000000
MSHR5848	Australia	AVAJ01000000
MSHR5858	Australia	AVAK01000000
MSHR668	Australia	CP000570, CP000571
NCTC13179	Australia	NC_022658, NC_022659
Pasteur52237	Vietnam	AAHV02000000
PB08298010	Arizona, USA	ARZO01000000
PHLS14	Philippines	ABBJ01000000
PHLS9	Pakistan	ABBL01000000
S13	Singapore	AAHW02000000
T4	Australia	JPNO01000000
VEL	Malaysia	APLI01000000

We detected the *Yersinia*-like fimbrial (YLF) gene by using CLC Genomics Workbench version 8.0.3 software, YP_110141.1, and blastn for each genome (CLCbio, Aarhus, Denmark) (*26*). ITS typing involved 3 sequence types: type C (FJ981718.1), type G (FJ981723.1), and type E (FJ981706.1) These types were queried against each target genome by using blastn version 2.2.31+ software (https://www.ncbi.nlm.nih.gov/news/06-16-2015-blast-plus-update/). Matches with 100% sequence alignment and >99% nt identity were assigned ITS types, and these blastn alignments were manually visualized/verified by using CLC Genomics Workbench version 8.0.3 software (*8*,*9*,*26*).

## Results

A dendrogram based on maximum-parsimony from analysis of core SNPs showed a distinct clade for genomes with an origin in the Western Hemisphere that branches off a node in common with genomes from isolates associated with Africa (Figure). BLAST analysis yielded ITS type G for isolates with a clear origin in the Western Hemisphere (Table 1). We also observed presence of the YLF gene in all isolates in our panel.

**Figure:**
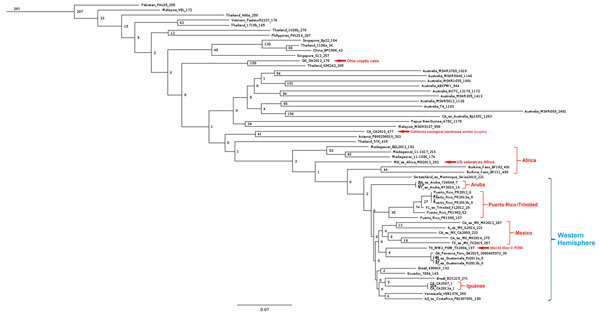
Dendrogram based on core SNP analysis of *Burkholderia pseudomallei* genomes by using maximum-parsimony for isolate kSNP3.021. Values at nodes indicate SNP distances between connecting nodes. Numbers to the right of the strain names are numbers of SNPs unique to that genome. Scale bar indicates nucleotide substitutions per site. POW, prisoner of war; SNP, single-nucleotide polymorphism.

Within the Western Hemisphere clade, distinct subclades appear to be associated with geographic origin (Figure). The largest subclade is the Puerto Rico/Trinidad clade, which consists of 5 genomes (PR1982, PR1998, PR2012, PR2013a, and PR2013b) from clinical cases and environmental isolates from Puerto Rico and 1 clinical case in Florida, USA (FL2012), from a resident of Trinidad (*2*,*20*,*27*). PR2013a and PR2013b, which were isolated from the same soil sample, also had no common SNPs detected but had 9 SNPs when compared with PR2012.

The second largest subclade consists of 5 genomes (CA2009, IL2014, MX2013, MX2014, and TX2015), which are all associated with clinical cases of melioidosis in patients who resided in or visited Mexico before seeking treatment in the United States (*2*,*27*,*28*). The patient from whom isolate MX2013 was obtained also had prior military service in Vietnam.

A subclade containing 4 genomes was resolved and consisted of GA2015, RI2013a, RI2013b, and TX2004. GA2015 was obtained from a patient who sought treatment in Georgia, USA, and resided in Panama but who had also visited Peru. The 2 isolates RI2013a and RI2013b, which have different colony morphologies, were obtained from a patient who traveled from Rhode Island, USA, to Guatemala. No common SNPs were detected for RI2013a and RI2013b. TX2004 was obtained from a resident of Texas, USA, who had spent time in Southeast Asia during World War II as a prisoner of war (*29*).

Genomes (NY2010 and 724644) from 2 cases of melioidosis in persons from the United States who traveled to Aruba clustered together and had 22 SNP differences between each other (*2*,*27*,*30*). Two isolates from pet iguanas (*Iguana iguana*) in California, USA (CA2007 and CA2013a) also clustered together (*31*). Three SNPs differentiate CA2007 from CA2013a.

Other genomes from isolates investigated in the United States from patients with no clear history of travel to regions to which melioidosis is endemic did not cluster within the Western Hemisphere clade. CA2010 groups with isolate PB08298010 in a subcluster among reference genomes from Southeast Asia and has ITS type C. OH2013 has ITS type CE and clusters most closely to K96243, which groups with other isolates from Thailand and Southeast Asia (*32*). Isolate MD2013, which was obtained from a US military veteran who served in Asia but most recently resided in Africa, has a genome that groups between isolates from Burkina Faso and Madagascar and has ITS type C.

## Discussion

Previous methods have been used to associate genetic features of *B. pseudomallei* with geographic origin. For example, it has been observed that the YLF gene cluster is predominant in strains from outside Australia, and our results are consistent with that association (*5*,*9*). We also observed that ITS type G predominates in our panel of isolates from the Western Hemisphere, which is consistent with results from our previous study (*5*). MLST has been used to associate isolates with geographic origin (*6*). However, MLST alone does not appear to be sufficient to provide geographic correlation when investigating an isolate of unknown provenance and can be confounded by homoplasy (*4*,*5*,*7*).

Analysis of SNPs in whole-genome sequences appears quite promising as a way to correlate isolates with geographic origin (*4*,*10*,*11*). The ability to link a strain with a geographic origin has been shown to be increasingly useful, especially in areas such as the US mainland, which so far does not appear to be endemic for melioidosis (*2*). This ability is particularly useful in cases in which there is no clear travel history to regions to which *B. pseudomallei* is endemic, or which may have resulted from exposure to contaminated products, such as medical supplies, from those areas (*3*). Our study supports previous observations by Sarovich et al. (*10*) and Chewapreecha et al. (*11*) that, on the basis of SNP analysis, isolates from the Americas belong to a distinct clade that branches off from strains from Africa. The larger size of our panel of isolates from the Western Hemisphere enabled a finer-scale association of some isolates on the basis of geographic origins and provided insights on isolates recovered from cases with no clear sources of exposures.

Isolates from Puerto Rico and the isolate from the patient who resided in Trinidad resulted in a clear association, which is surprising because Trinidad is much closer to Venezuela than to Puerto Rico. Isolates PR2013a and PR2013b were obtained from the same soil sample and do not have common SNPs. The soil sample from which PR2013a and PR2013b was isolated was <250 m from the residence of the patient who was infected with isolate PR2012. Not only do the soil isolates cluster with PR2012, but there were only 9 SNPs differences between them and PR2012, which supports the supposition that the area close to the home of the patient was the source of infection. This supposition is analogous to the observation in a case in Australia where only 3 SNPs separated a clinical isolate from an environmental isolate obtained near the home of the patient (*12*).

The association among isolates from patients with links to Mexico was also clear on the basis of clustering in the phylogenetic tree. However, each isolate is relatively distant from each other (e.g., 3606 SNPs distinguish MX2014 from IL2012), which indicates that more genetic diversity has been captured for isolates with origins in Mexico than for those with origins in Puerto Rico.

The subclade consisting of GA2015, RI2013a, RI2013b, and TX2004 appears to be related to the subclade from Mexico. Also, no common SNPs were detected for RI2013a and RI2013b despite difference in colony morphotypes. This finding is consistent with a recent observation in another strain in which no common SNPs were detected among genomes of a variety of colony morphotypes, which indicated that the differences were not caused by mutations, but possibly by differential gene expression or another mechanism (*33*).

TX2004 is an isolate obtained from a patient who has been reported as having the longest incubation period for melioidosis. This patient was a US military veteran who was captured during World War II by the Japanese and interned in various camps in Southeast Asia as a prisoner of war; he did not have a travel history to other regions to which *B. pseudomallei* is endemic. His case of melioidosis was attributed to exposure to *B. pseudomallei* 62 years before development of symptoms (*29*). However, we found that TX2004 belongs to the Western Hemisphere clade and groups with genomes from isolates from melioidosis patients who had travel histories to Guatemala, Panama, and Peru. This finding, and the fact that TX2004 is ITS type G, suggests that TX2004 might not have been acquired by the patient in the Pacific theater during World War II.

For the 2 isolates obtained from travelers to Aruba, we found no epidemiologic link between the 2 cases (NY2010 and 724644) other than travel to Aruba for vacation. This finding supports the hypothesis that Aruba was the source of exposure and highlights the usefulness of well documented travel records for epidemiologic associations.

We observed clustering of genomes from isolates obtained from 2 green iguanas. The first case (CA2007) was reported in 2007 in northern California, and the iguana died ≈2.5 years later. The second case (CA2013a) was reported in a 1.6-year-old iguana in southern California. The iguanas were purchased from different pet stores and the cases were separated in time. Thus, there was no clear epidemiologic link between the cases. No background information was available on the origin of the iguanas or the pet store suppliers. Both iguanas were not transported after purchase and were confined to the homes of the owners.

Earlier MLST data indicated that both of these isolates had ST518, which had also been seen in isolate PB1007001, obtained from a tourist from Arizona, USA, in whom melioidosis developed after this person was infected in Costa Rica. This finding suggested that the iguanas were infected in Central America before importation and is consistent with the known range of iguanas (Florida to southern Brazil) and the observation that most iguanas imported into the United States come from Central America (*31*,*34*,*35*). The genomes from the 2 isolates are highly related (only 3 common SNPs) and suggests that the iguanas originated in the same region. The same breeding facility could also be involved, assuming that the iguanas were farm raised.

The association of clades with geographic origin promises to be a useful tool when investigating cases of melioidosis in patients without a clear exposure history, such as travel to multiple countries to which this disease is endemic or for patients with no known travel history. Isolate MD2013 was obtained from a retired person from the United States who resided in Ghana and traveled around Africa. He also had previous military service in Asia, although not in regions with a strong history of melioidosis. He sought treatment in the District of Columbia and Maryland, USA, area after symptoms developed. MD2013 has a genome that groups between isolates from Burkina Faso and Madagascar and supports infection with the bacteria in Africa. The grouping of MX2013 with other genomes from Mexico indicates infection occurred in Mexico instead of Vietnam. An isolate from a case in a zoological warehouse worker without a travel history to areas to which melioidosis is endemic has a genome (CA2010) that does not belong within the clade for isolates from the Western Hemisphere. This isolate belongs within a subclade that contains an isolate (PB08298010) from a resident of Arizona who also did not have a clear travel history (*36*). We previously reported that PB08298010 was ITS type G on the basis of an earlier draft genome sequence, but a more recent analysis of a higher-quality whole-genome sequence indicates it has ITS type CE (A. Tuanyok, 2015, pers. comm.) (*5*). The ITS type CE and the location of this subclade support an origin outside the Western Hemisphere, more likely in Asia, which supports the earlier proposal that PB08298010 might have resulted from exposure to contaminated medical supplies from Southeast Asia (*10*,*36*). A similar situation might also be true for a fatal case in a patient in Ohio, USA, who had no travel history outside the continental United States (*32*). Isolate OH2013 groups with isolates from Thailand and is clearly not associated with those in the Western Hemisphere.

The genome sequences analyzed in this study will assist future epidemiologic investigations of melioidosis, especially in cases potentially associated with exposure in the Western Hemisphere. We believe that, as more genome sequences become available, the robustness and resolution of this analysis will improve and thus enhance the ability to associate isolates of *B. pseudomallei* with geographic origin.
